# Consider Mitochondrial DNA Rearrangements as a Cause of Idiopathic Pancreatitis

**DOI:** 10.5152/tjg.2025.24704

**Published:** 2025-01-06

**Authors:** Josef Finsterer, Sounira Mehri

**Affiliations:** 1 Department of Neurology, Neurology and Neurophysiology Center, Vienna, Austria; 2 Biochemistry Laboratory, LR12ES05 Nutrition-Functional Foods and Vascular Health Faculty of Medicine, Monastir, Tunisia

We were interested to read the article by Baş et al^1^ on a study about the contribution of sequence and copy number variants to the pathophysiology of idiopathic pancreatitis in Turkish patients. Next-generation sequencing and multiple ligand-dependent probe amplification of the *PRSS1, SPINK1, CTRC*, and *CFTR* genes revealed that sequence variants of potential clinical significance were found in *PRSS1* in 13%, in *SPINK1* in 6%, in *CTRC *in 5%, and in *CFTR* in 27%.^1^ No copy number variants were detected in any of these 4 genes.^1^ It was concluded that 43% of participants had a potential genetic risk factor for idiopathic pancreatitis and that genetic variants were more prevalent in early onset disease than in late-onset disease.^1^ The study is stimulating, but some points should be discussed. Information on informed consent was not required as the manuscript does not contain original data.

The first point is that mitochondrial DNA (mtDNA) has not been tested for sequence variants. Since pancreatitis can be a phenotypic feature of mitochondrial disorders (MIDs), either as an isolated disease^2^ or as a disease together with other organ manifestations,^3^ it is recommended to test mtDNA for sequence variants in all patients with idiopathic pancreatitis. Not only patients with clinical features of a hereditary disease and a positive family history should be examined, but also individual cases so that spontaneous mutations are not overlooked. Pancreatitis has been reported in both syndromic and non-syndromic MIDs. Pancreatitis occurs in particular in Pearson syndrome, Kearns–Sayre syndrome, myclonic epilepsy with ragged-red fibers (MERRF) syndrome, and mitochondrial encephalopthy, lactic acidosis, and stroke-lik episodes (MELAS) syndrome. It would therefore also have been interesting to examine all patients for anemia or pancytopenia, as single mtDNA deletions can manifest phenotypically as Pearson syndrome (pancytopenia plus pancreatitis).^4^ The second point is that the pathogenicity of the discovered variants has not been analyzed. No biochemical, segregation, or functional studies were performed to assess whether the discovered variants are actually responsible for pancreatitis. In order to establish a causal link between the discovered variants and pancreatitis, it would have been imperative to also establish or exclude a causal connection. This is important in order to know whether or not these patients need to be further investigated for other possible causes of pancreatitis.

The third point is that first-degree relatives of the 68 included patients were not screened for subclinical pancreatitis. Although it is important to take a family history, it is even more important to examine clinically ill and non-ill family members, as the disease may only manifest itself in them as an increase in pancreatic enzymes or mushy stools.

The fourth point is that there is no discussion about circulating mtDNA as a biomarker for severe acute pancreatitis. In a recent study, circulating mtDNA was shown to be a marker for early prediction of the severity of acute pancreatitis.^5^ However, circulating mtDNA is also elevated in various other inflammatory diseases. Since circulating mtDNA can also trigger an inflammatory response in pancreatic necrosis, it would also have been interesting to determine the circulating mtDNA level in the included patients.

In summary, it can be said that this study has limitations that relativize the results and their interpretation. Addressing these limitations could strengthen the conclusions and reinforce the message of the study. When screening patients with idiopathic pancreatitis for possible underlying causes, it is recommended that they also be tested for mtDNA variants. It is also recommended to screen first-degree relatives of patients with idiopathic pancreatitis for subclinical or mildly manifest pancreatitis.

## Data Availability

The data that support the findings of this study are available on request from the corresponding author.
